# Spinal adhesive arachnoiditis in an adult patient with spinal muscular atrophy type 3 treated with intrathecal therapy

**DOI:** 10.1186/s12883-024-03543-0

**Published:** 2024-01-24

**Authors:** Jakub Ubysz, Magdalena Koszewicz, Joanna Bladowska, Slawomir Budrewicz

**Affiliations:** 1 Department of Neurology, Wroclaw Medical University, Borowska 213, Wroclaw, 50-556 Poland; 2 Department of Preclinical Sciences, Pharmacology and Medical Diagnostics, Faculty of Medicine, Wroclaw University of Science and Technology, Wroclaw, Poland; 3 Department of Radiology, Wroclaw 4th Military Hospital, Wroclaw, Poland

**Keywords:** Spinal muscular atrophy, Spinal adhesive arachnoiditis, Intrathecal therapy, MRI

## Abstract

**Background:**

Spinal adhesive arachnoiditis is a chronic inflammatory process of the leptomeninges and intrathecal neural elements. The possible causes of arachnoiditis are: infections, injuries of spinal cord, surgical procedures and intrathecal administration of therapeutic substances or contrast.

**Case presentation:**

We present a case of 56-old woman with spinal muscular atrophy type 3 who developed a severe back pain in the lumbosacral region after the fifth dose of nusinersen given intrathecally. Magnetic resonance of lumbosacral spine showed spinal adhesive arachnoiditis. She received high doses of methylprednisolone intravenously, and later non-steroidal anti-inflammatory drugs, alpha lipoic acid, vitamins and rehabilitation with slight improvement.

**Conclusions:**

The authors summarize that scheduled resonance imaging of the lumbosacral spine may be an important element of the algorithm in the monitoring of novel, intrathecal therapy in patients with spinal muscular atrophy.

## Background

Spinal adhesive arachnoiditis (SAA) is an inflammatory process of the arachnoid membrane which encases nerve roots. The possible etiologic factors of SAA include infections, spinal cord injury, spine surgery and intrathecal administration of contrast agents or therapeutic substances [1, 2]. Diagnosis of SAA is made based on clinical presentation and MRI. SAA treatment is difficult and often ineffective. Conservative therapy involves high-dose corticosteroids and nonsteroidal anti-inflammatory drugs [3].

## Case presentation

The patient gave her written informed consent for the case publication, and has been offered the opportunity to review the manuscript before submission.

**Fig. 1:**
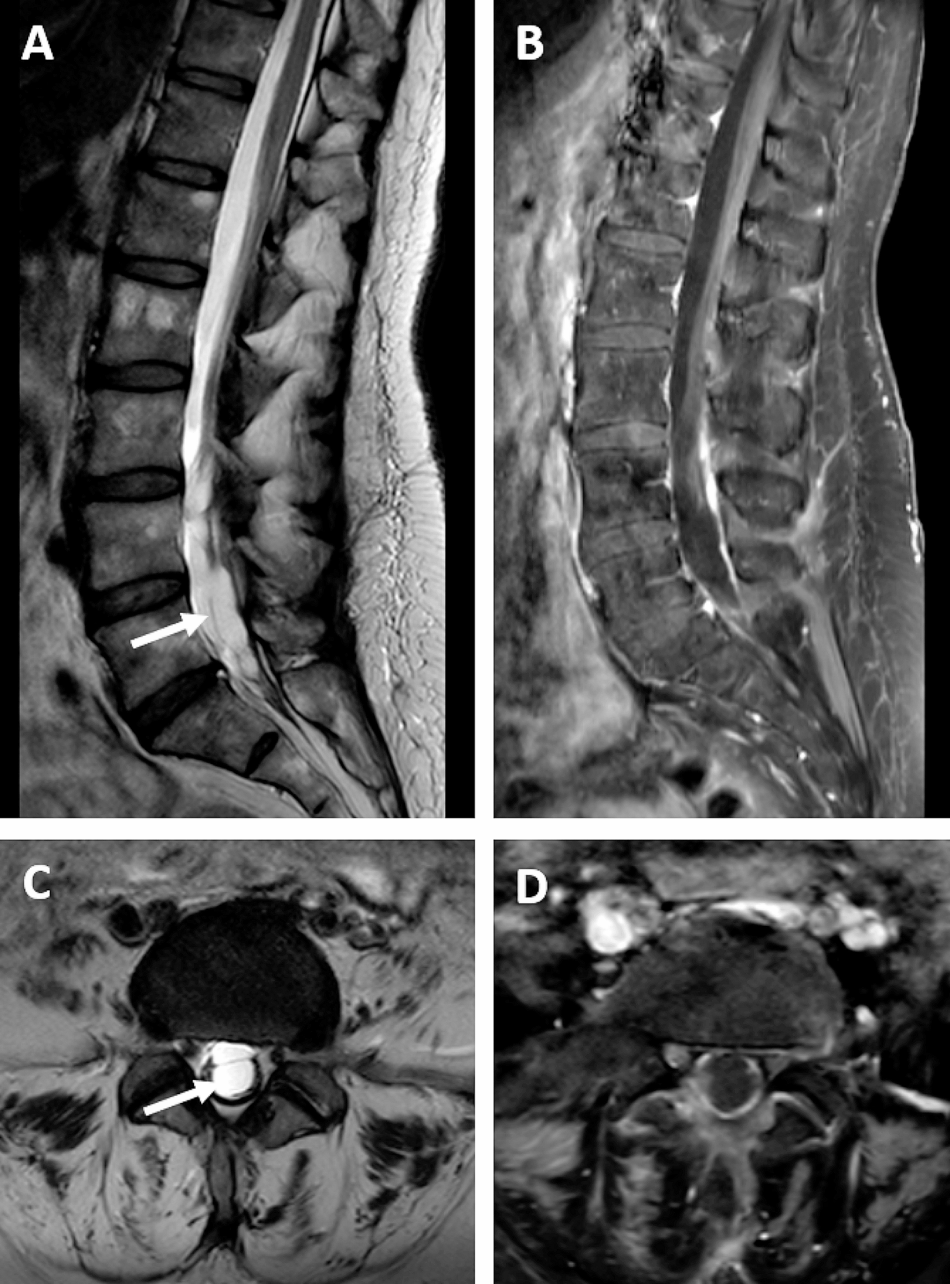
MRI of the lumbosacral spine performed on a 3T scanner, T2-weighted (**A**, **C**) and T1-weighted images after contrast administration (**B**, **D**). MR examination revealed the distorted nerve roots, adherent to the thecal sac, creating the “empty thecal sac” sign (arrow) typical for type II arachnoiditis

A 56-year-old woman with spinal muscular atrophy (SMA) type 3, who had been treated with intrathecal administration of nusinersen for 9 months, was admitted to the Neurological Department with severe back pain in the lumbosacral region radiating to her left leg. The pain started after she received the last (fifth) dose of nusinersen. She never had lumbar puncture before starting the intrathecal treatment, nor any infections, injuries or surgical interventions. Neurological examination revealed increased paravertebral muscle tone and walking deterioration due to pain. Otherwise, the neurological state was unchanged compared to the examination performed three months earlier. In particular, no sensory disturbance, sphincter dysfunction or Lasegue’s sign were found. MRI of lumbosacral spine showed that the nerve roots were distorted and adherent to the thecal sac, creating the characteristic “empty thecal sac” sign. Enhancement of the nerve roots was observed following the administration of contrast. MRI also demonstrated changes of paravertebral soft tissues in the region of previous lumbar punctures (Fig. 1). Erythrocyte sedimentation rate was mildly elevated (14 mm), high sensitivity C-reactive protein level was normal. Cerebrospinal fluid analysis was within the normal range. Based on the symptoms and the MRI, the patient was diagnosed with adhesive arachnoiditis. Initially, she received high doses of methylprednisolone intravenously (1 g daily for 5 days) and galantamine in intramuscular injections. She was prescribed non-steroidal anti-inflammatory drugs (diclofenac, indomethacin), alpha lipoic acid, vitamins (B1, B2, B6, D and E) and rehabilitation. Six and twelve weeks later, the patient reported persistent back pain of lower intensity without radiation to the left leg. MRIs were similar to the previous one. As a consequence the patient discontinued the therapy. The described adverse reaction was reported, in accordance with the procedure, to the local pharmacovigilance authorities, and to the pharmaceutical company.

## Discussion and conclusion

SMA is a rare neuromuscular disease caused by mutation of the *SMN1* gene leading to progressive weakness and atrophy of muscles. Nusinersen was approved by US Food and Drug Administration in December 2016 and is the first treatment for SMA [4]. It is administered as an intrathecal bolus injection by lumbar puncture with 4 loading doses and then once every 4 months. The most common adverse reactions are associated with the lumbar puncture procedure (e.g. headache, back pain) [5]. SAA has not been reported after intrathecal administration of nusinersen yet [6]. Several intrathecal medical substances were described as potential etiological factors of SAA with one of the major causes being the contrast agents introduced into the subarachnoid space for myelography. Intrathecal administration of amphotericin B, methotrexate, anesthetic agents and steroids has also been reported to provoke inflammation of the arachnoid membrane [1].

The clinical manifestation ranges from subclinical to advanced forms of the disease. In many cases it can be asymptomatic and remain undiagnosed. The true incidence of SAA is therefore hard to determine and is reportedly underestimated [1, 2, 7].

Due to the inflammatory process, the nerve roots become adherent to each other and to the thecal sac. On the basis of the characteristic morphological appearance on MR imaging, SAA has been divideed into three types. In type I, the nerve roots are clumped together and form a central conglomeration. In type II, the nerve roots are distorted and adherent to the thecal sac, creating the “empty thecal sac” sign as we present in our case. In type III, a large central soft-tissue mass fills the thecal sac as a result of the nerve roots clumping up with the thecal sac [8].

In the presented case, the most probable cause of SAA are the repeated lumbar punctures combined with the drug administration. The advanced vertebral column deformities in the course of SMA could additionally lead to creating favorable conditions for SAA development. SAA may be secondary to abnormal anatomical conditions of the spine, as is seen in patients with SMA, sometimes with very significant severity. Activation of inflammatory processes, primary and secondary, with subsequent ischaemia and scarring of spinal canal structures, also appears to be important in the pathogenesis of SAA. Genetic predisposition to the formation of abnormal fibrinolytic scars should be taken into account in the development of SAA [1, 2, 9]. The authors also consider the possibility of a generalized inflammatory process with activation of systemic pro- and anti-inflammatory factors, which, however, requires further study.

In the conclusion the authors indicated that spinal adhesive arachnoiditis may be a possible, significant adverse reaction after intrathecal administration of nusinersen. Scheduled resonance imaging of the lumbosacral spine may be an important element of the algorithm in therapy monitoring and may allow the diagnosis of early forms of SAA.

## Data Availability

No datasets were generated or analysed during the current study.

## Abbreviations

MRI: magnetic resonance imaging
SAA: spinal adhesive arachnoiditis
SMA: spinal muscular atrophy
SMN: survival motor neuron
